# Prescription of glucagon-like peptide 1 agonists and risk of subsequent open-angle glaucoma in individuals with type 2 diabetes mellitus

**DOI:** 10.7150/ijms.90273

**Published:** 2024-01-12

**Authors:** Chih-Chun Chuang, Kai Wang, Chao-Kai Chang, Chia-Yi Lee, Jing-Yang Huang, Heng-Hsiung Wu, Po-Jen Yang, Shun-Fa Yang

**Affiliations:** 1 Institute of Medicine, Chung Shan Medical University, Taichung, Taiwan.; 2 Department of Ophthalmology, Changhua Christian Hospital, Changhua, Taiwan.; 3 Department of Post-Baccalaureate Medicine, College of Medicine, National Chung Hsing University, Taichung, Taiwan.; 4 Department of Ophthalmology, Cathay General Hospital, Taipei, Taiwan.; 5 Departments of Ophthalmology, Sijhih Cathay General Hospital, New Taipei City, Taiwan.; 6 School of Medicine, College of Medicine, Fu Jen Catholic University, New Taipei, Taiwan.; 7 Department of Ophthalmology, Nobel Eye Institute, Taipei, Taiwan.; 8 Department of Optometry, Da-Yeh University, Chunghua 515, Taiwan.; 9 Department of Ophthalmology, Jen-Ai Hospital Dali Branch, Taichung, Taiwan.; 10 Department of Medical Research, Chung Shan Medical University Hospital, Taichung, Taiwan.; 11 Program for Cancer Biology and Drug Discovery, China Medical University, Taichung, Taiwan.; 12 Department of Family and Community Medicine, Chung Shan Medical University Hospital, Taichung, Taiwan.; 13 School of Medicine, Chung Shan Medical University, Taichung, Taiwan.

**Keywords:** glucagon-like peptide 1 receptor agonist, open angle glaucoma, epidemiology, age, vascular

## Abstract

**Background:** The glucagon-like peptide 1 receptor agonist (GLP-1RA) is an antidiabetic medication with vascular protection and anti-inflammatory properties. Theoretically, the use of GLP-1RA should inhibit the development of open-angle glaucoma (OAG) as both vascular damage and inflammation are associated with OAG. Therefore, our objective was to investigate the association between the application of GLP-1RA and the subsequent OAG in individuals with type 2 diabetes mellitus (T2DM).

**Methods:** We conducted a retrospective cohort study by using data from the National Health Insurance Research Database (NHIRD) of Taiwan. Participants with T2DM were divided into those who used GLP-1RA and those who did not, forming the GLP-1RA and control groups. The primary outcome was the occurrence of OAG based on diagnostic codes. Cox proportional hazard regression was employed to calculate the adjusted hazard ratio (aHR) and 95% confidence interval (CI) for OAG.

**Results:** 91 patients in the control group developed OAG, and 40 patients in the GLP-1RA group developed OAG. After adjustment for all covariates, the GLP-1RA group exhibited a significantly lower incidence of OAG compared with the control group (aHR: 0.712, 95% CI: 0.533-0.936. P = 0.0025). In the subgroup analyses, the association between GLP-1RA use and OAG incidence was more pronounced in patients with T2DM using GLP-1RA and aged younger than 60 years (P = 0.0438).

**Conclusion:** The prescription of GLP-1RA is associated with a lower incidence of subsequent OAG in individuals with T2DM, and this association was more significant in patients with T2DM under the age of 60 years.

## Introduction

Type 2 diabetes mellitus (T2DM) is characterized by insulin resistance, resulting in hyperglycemia 1. In advanced stages, T2DM can result in several comorbidities such as acute myocardial infarction, cerebrovascular disease, and diabetic retinopathy 2, 3. Current management strategies for T2DM primarily involve the use of antidiabetic medications, with insulin injection reserved for more severe cases 4. Among the antidiabetic medicines, glucagon-like peptide 1 receptor agonists (GLP-1RA) have been demonstrated to effectively reduce glycated hemoglobin levels by 2% 5.

In addition to its antihyperglycemic effects, GLP-1RA exhibits protective effects on various other organs 5. The use of GLP-1RA has been shown to preserve the kidney function in T2DM patients 6, 7, and the use of GLP-1RA can also reduce the incidences of all-cause mortality, major adverse cardiovascular event and obesity 8, 9. Studies have indicated that GLP-1RA can influence the development and progression of various ocular diseases, particularly those featuring neurosensory impairment 10, 11. An experimental study demonstrated that the application of GLP-1RA can mitigate neurodegeneration in the retina 11. GLP-1RA is associated with a reduction in the incidence of diabetic retinopathy as well as a decrease in retinal vascular leakage and damage to the blood-retinal barrier 12, 13. However, another study reported a nonsignificant association between the use of GLP-1RA and retinal angiogenesis 10. Additionally, patients with T2DM using GLP-1RA or sodium-glucose transport protein 2 inhibitors exhibit a significantly lower rate of dry eye disease than those using other antidiabetic medications such as metformin monotherapy 14, 15. However, evidence regarding the relationship between GLP-1RA use and the incidence of open-angle glaucoma (OAG) is limited. Given the vascular-protective effects of GLP-1RA and the relationship between OAG and impaired ocular vasculature 16, 17, a relationship between GLP-1RA use and OAG incidence may exist.

This study investigated the relationship between GLP-1RA use and the incidence of OAG. The other risk factors for OAG development were also adjusted for in the analysis model.

## Materials and Methods

### Data Source

The current study adhered to the principles of the Declaration of Helsinki as revised in 1964 and its subsequent amendments. This study was approved by both the National Health Insurance Administration of Taiwan and Institutional Review Board of Chung Shan Medical University (Project code: CS1-20108). The requirement for written informed consent was waived by both institutions. We used data from the Taiwan National Health Insurance Research Database (NHIRD), which contains the data of approximately 23 million individuals in Taiwan for the period from January 1, 2015, to December 31, 2020. The accessible data in this Taiwan NHIRD include *International Classification of Diseases-Ninth Revision* (*ICD-9*) diagnostic codes, *International Classification of Diseases-Tenth Revision* (*ICD-10*) diagnostic codes, age, sex, income level, education level, urbanization level, occupation type, laboratory examination codes, medical department codes, surgery codes, image examination codes, procedure codes, and the international Anatomical Therapeutic Chemical (ATC) codes for medicines.

### Participant Selection

**Figure 1:**
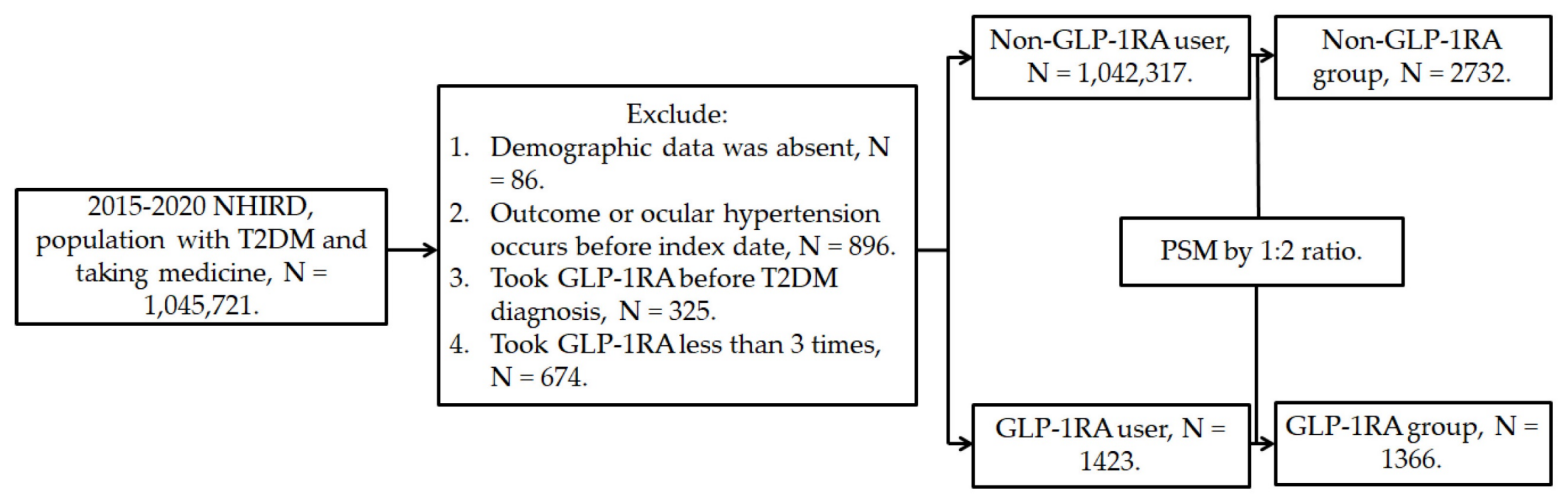
** Flowchart of participant selection.** NHIRD: National Health Insurance Research Database, N: number, T2DM: type 2 diabetes mellitus, GLP-1RA: glucagon-like peptide 1 receptor agonist, PSM: propensity score-matching.

This retrospective population-based cohort study identified individuals with T2DM who used GLP-1RA based on the following criteria: (1) a diagnosis of T2DM according to *ICD-9* and *ICD-10* codes from 2015 to 2019, (2) follow-up appointments in either family medicine or internal medicine departments for more than 3 months based on department codes, and (3) the prescription of GLP-1RA, including exenatide and liraglutide, according to the relevant ATC codes. The index date was set 6 months after the initiation of GLP-1RA treatment. Furthermore, the following exclusion criteria were applied to enhance the homogeneity of the study population: (1) absence of demographic data, (2) presence of glaucoma or ocular hypertension before the index date, (3) use of GLP-1RA before the T2DM diagnosis, and (4) fewer than three prescriptions of GLP-1RA. For comparison, each individual with T2DM using GLP-1RA was matched to two individuals with T2DM who did not use GLP-1RA, and the latter group of individuals served as the control group. The propensity score-matching (PSM) method was adopted to match the two groups through the adjustment of demographic data, systemic disease factors, and medicine covariates. Following the selection process, a total number of 1366 and 2732 participants with T2DM were included in the GLP-1RA group and the control group, respectively. The patient selection flowchart is presented in Figure 1.

### Primary Outcome

The primary outcome in this study was the incidence of newly developed OAG, and it was determined on the basis of the following criteria: (1) diagnosis of OAG according to related *ICD-9* and *ICD-10* diagnostic codes; (2) use of slit-lamp biomicroscopy and fundoscopic examinations before or at the same time as OAG diagnoses, as indicated by procedure codes; (3) use of optical coherence tomography or visual field examinations before or at the same time as OAG diagnoses, identified through examination codes; (4) use of topical or systemic antiglaucomatous medications after receiving the diagnosis of OAG, as documented using ATC codes; and (5) OAG diagnoses confirmed by an ophthalmologist. The individuals with T2DM in the current study were followed up until one of the following conditions occurred: (1) incidents of OAG, (2) participant withdrawal from the National Health Insurance program, or (3) the end of the NHIRD data collection period on December 31, 2020.

### Confounders

In our statistical analysis, we accounted for various demographic factors, systemic comorbidities, and medications to control for confounders that might influence the incidence of OAG: age, sex, urbanization level, hypertension, hyperlipidemia, ischemic heart diseases, ischemic stroke, hemorrhagic stroke, peripheral vascular disease, kidney disease, as well as medications such as sulfonylureas, biguanides which indicates the metformin, thiazolidinediones, alpha glucosidase inhibitors, dipeptidyl peptidase-4 inhibitor, insulin, statin and corticosteroids. These factors were identified based on *ICD-9* and *ICD-10* diagnostic codes as well as ATC codes in the NHIRD. The number of systemic diseases was incorporated into the adapted diabetes complications severity index (aDCSI) by using difference scores. To ensure that these confounders sufficiently influenced the risk of OAG, only factors with a disease or prescription interval exceeding 2 years prior to the index date were included in statistical analyses. Because nearly all the GLP-1RA users in our study were concurrently taking metformin as their antidiabetic treatment (~93%), the resulting high collinearity between GLP-1RA and metformin led to prominent statistical bias or error in the initial statistical analysis. Consequently, we did not include metformin as a covariate in our final statistical analysis.

### Statistical Analysis

Statistical analyses were performed using SAS version 9.4 (SAS Institute Inc, Cary, NC, USA). Descriptive analyses were used to present basic demographic information, aDCSI, and related medications for both the groups. The absolute standardized difference (ASD) was calculated to compare the differences between the two groups, with an ASD >0.1 considered to be statistically significant. Following descriptive analyses, Cox proportional hazard regression was used to calculate the adjusted hazard ratios (aHR) with corresponding 95% confidence intervals (CIs) for determining the OAG incidence between the GLP-1RA group and control group. The demographic features, systemic morbidities, and medications were all adjusted for in the Cox proportional hazard regression. In subgroup analyses, patients with T2DM were categorized by age and sex, and the Cox proportional hazard regression was applied again to compare the aHR and 95% CI for OAG among different subgroups. Furthermore, interaction tests were conducted to illustrate the influence of GLP-1RA on OAG development in different subgroups. Statistical significance was set at P < 0.05, and a P value of <0.0001 was presented as P < 0.0001.

## Results

Table 1 presents the basic characteristics of the GLP-1RA users and non-GLP-1RA users. The distribution of age and sex was similar between the two groups, which was due to PSM. Additionally, the urbanization level and the distribution of aDCSI were similar (ASD < 0.1). Regarding medications, a higher proportion of GLP-1RA users had insulin application than non-GLP-1RA users (36.44% versus 5.21%; ASD: 0.2148). However, the ratios of other medications were similar between the two groups (all ASD < 0.1; Table 1).

Over the entire follow-up period, 91 and 40 patients had OAG in the non-GLP-1RA and GLP-1RA groups, respectively. The GLP-1RA group exhibited a significantly lower incidence of OAG than the control group after adjustment for all the confounders (aHR: 0.712, 95% CI: 0.533-0.936. P = 0.0025; Table 2). In subgroup analyses, patients with T2DM taking GLP-1RA, regardless of age (both younger and older than 60 years), exhibited a significantly lower incidence of OAG than the non-GLP-1RA users (both upper limits of 95% CI lower than 1). Moreover, the association between GLP-1RA use and OAG incidence was more pronounced in the patients with T2DM taking GLP-1RA and those aged younger than 60 years (P = 0.0438). However, the association between GLP-1RA use and OAG incidence did not significantly differ between sex subgroups (P = 0.5621; Table 3).

## Discussion

The current study revealed an association between the use of GLP-1RA and a lower incidence of OAG in individuals with T2DM compared with those not taking GLP-1RA. Notably, this association was more pronounced in patients with T2DM aged ≤60 years. Furthermore, the association between GLP-1RA and OAG development was consistent across different sexes in individuals with T2DM.

GLP-1RA has various beneficial various functions in addition to its antihyperglycemic ability 18, 19. A previous study demonstrated the cardiovascular protective effects of GLP-1RA according to a lower incidence of cardiovascular death in individuals with T2DM patients under GLP-1RA treatment 20. Additionally, the incidence rates of nonfatal myocardial infarction and cerebrovascular diseases were significantly lower in individuals using GLP-1RA 21. The GLP-1RA has been demonstrated to exert renal protective effects through the amelioration of albumin excretion and the prevention of creatinine elevation 22-24. Moreover, GLP-1RA has the potential for use in managing Alzheimer's disease and Parkinson's disease 25. At the molecular level, the cardiovascular protection offered by GLP-1RA may be due to its anti-inflammatory properties and antioxidative effects 16, 22, 26. Glaucoma, including OAG, is a neurodegenerative disease characterized by the death of retinal ganglion cells 27, 28. It shares protein features with other neurodegenerative diseases such as Alzheimer's disease 29. Although intraocular pressure is a crucial risk factor for OAG 30, the impairment of ocular vasculature has recently been recognized as another major factor contributing to OAG development 31. In individuals with OAG, ocular blood flow decreases and can be reversed by dorzolamide 32. Additionally, the vascular densities of the optic disc and macula are significantly lower in individuals with OAG 33, 34. Furthermore, diseases that damage vasculature, such as hypertension, are significant risk factors for OAG 28. GLP-1RA can generally preserve vasculature and suppress inflammation, which are both predisposing factors for OAG 28, 31. Experimental glaucoma models have shown that GLP-1RA suppresses interleukin-1α production, tumor necrosis factor α production, astrocyte transformation, and the subsequent death of retinal ganglion cells 35. Additionally, topical application of GLP-1RA has been found to reduce extracellular glutamate expression, preventing retinal neurodegeneration, including glial activation and neural apoptosis 11. Consequently, we infer that the use of GLP-1RA could alter the incidence of subsequent OAG. This inference aligns with the results of the current study.

The current study revealed an association between the use of GLP-1RA and a lower incidence of OAG in individuals with T2DM. Although the potential protective effect of GLP-1RA on glaucoma development has been suggested 35, 36, research exploring this concept is limited. A previous study demonstrated the protective effect of GLP-1RA on the development of primary OAG, glaucoma suspect, and low-tension glaucoma; however, the specific effect of GLP-1RA on OAG was not evaluated 37. To our knowledge, this study provides preliminary evidence of the possible correlation between GLP-1RA application and the incidence of subsequent OAG in individuals with T2DM. Additionally, individuals with previous OAG and ocular hypertension were excluded to ensure that the OAG episodes in the current study occurred after the application of GLP-1RA treatment. Moreover, the effects of several known risk factors of OAG, including age, hypertension, and corticosteroid usage, were adjusted in the multivariable analysis in the current study 28, 30, and the diabetes duration were all between one to five years in both the GLP-1RA and control groups. Consequently, the application of GLP-1RA may serve as an independent protective factor for the development of subsequent OAG, possibly due to its vascular protection effects. Notably, T2DM itself is considered a risk factor for OAG 38, and severe T2DM is correlated with a higher incidence of OAG. In the current study, the percentage of antidiabetic medications was numerically higher in the GLP-1RA group, whereas the ratio of insulin prescription was significantly higher in the GLP-1RA group. This observation might suggest that the severity of T2DM was higher in the GLP-1RA group, which would theoretically be associated with a higher risk of OAG. However, the study found a reduced risk of OAG in this population after GLP-1RA prescription, implying a potential protective effect of GLP-1RA on OAG development. About the generalizability of our results, most of the Taiwanese are collateral to the Chinese ethnicity. Consequently, our result could be applied to the other nation that mainly consists of Chinese like the China and Singapore.

In the subgroup analyses, all age and sex subgroups with T2DM using GLP-1RA demonstrated a significantly lower risk of OAG development compared with the T2DM population not using GLP-1RA. This aligns with a study that demonstrated a reduced incidence of OAG, glaucoma suspect, and low-tension glaucoma in the T2DM population using GLP-1RA 37. The current study further supports these findings, indicating a consistent and universal effect of GLP-1RA on the development of subsequent OAG in T2DM populations across various age and sex subgroups. In particular, patients with T2DM aged younger than 60 years with GLP-1RA application exhibited a significantly lower risk of subsequent OAG development compared with their older counterparts. Because age is a well-established risk factor for OAG development 30, it is reasonable to expect that older individuals with T2DM exhibited a higher incidence of OAG. However, the aHRs for both age subgroups were not markedly different, suggesting that the observed difference may be clinically nonsignificant. Furthermore, the incidence of OAG after GLP-1RA prescription did not show a significant difference between the two sex subgroups. Although previous studies have proposed conflicting findings regarding the vulnerability of males or females to the development of OAG 27, 30, the results of the current study indicated that the incidence of OAG was not affected by the different sexes in the T2DM population.

The epidemiology of T2DM underscores its status as one of the most prevalent systemic diseases globally, with an overall prevalence exceeding 8% in a previous study 39. The presence of T2DM is associated with major morbidities, including ischemic heart disease, cerebrovascular accidents, diabetic kidney disease, and diabetic retinopathy 40. Among these complications, the mortality rate of patients with T2DM-associated complications can reach 60.2 per 100,000 individuals 41, and diabetic retinopathy accounts for 2.5% of legal blindness 42. OAG is also a prevalent ocular disease, affecting approximately 2.5% of the European population according to previous research 30. Moreover, advanced OAG is a major cause of legal blindness, contributing to 11% of cases, further contributing to a huge economic burden 42. Because both the T2DM and OAG are common diseases and can impair the vision to a large extent 27, 39, 42, management strategies that could reduce the possibility of OAG development in the T2DM population should be identified.

The current study has several limitations. First, the use of claims data as the primary data resource rather than real medical documents prevents the analysis of crucial information, including the initial blood sugar and glycated hemoglobin levels in patients with T2DM, subsequent blood sugar and glycated hemoglobin levels after the prescription of GLP-1RA and other antidiabetic medications, fundoscopic images of all individuals with T2DM, initial intraocular pressure among patients with OAG, results of optical coherence tomography and visual field examinations in patients with OAG, treatment outcomes of patients with OAG, and changes in intraocular pressure after anti-glaucomatous treatment in patients with OAG. Moreover, the retrospective design of the current study, despite the application of PSM, may reduce homogeneity compared with a study with a prospective design. We did not consider the metformin application as a covariate in the statistical analysis since the high collinearity between GLP-1RA and metformin (about 93% patients with GLP-1RA also took metformin) would cause prominent statistical bias/error even in interaction test or stratified analysis. However, the metformin is an important medication for T2DM control and the exclusion of metformin usage in the statistical analysis due to any reason will decrease the integrity of both data and results of the current study. Furthermore, misdiagnosis of OAG may occur as some patients with normal tension glaucoma could be included with the *ICD-9* or *ICD-10* diagnostic codes of OAG in clinical practice. Finally, the exclusion and matching process resulted in the loss of approximately 40% of individuals with GLP-1RA application. Although this was done to enhance diagnostic accuracy and homogeneity, the impact of patient loss might not be significant as the GLP-1RA group still included over 1000 patients, a number not inferior to previous studies 32, 34.

In conclusion, the use of GLP-1RA is associated with a lower incidence of subsequent OAG development in individuals with T2DM, even after adjustment for several risk factors for OAG. Furthermore, this association was more pronounced in patients with T2DM who were younger than 60 years. Consequently, GLP-1RA may be considered as a potential recommendation for patients with T2DM with known risk factors for OAG development. However, further large-scale prospective studies are essential to comprehensively evaluate the influence of GLP-1RA usage on glaucoma progression.

## Figures and Tables

**Table 1:** Clinical characteristics of the GLP-1RA users and the non-GLP-1RA population

Characteristic	Non-GLP-1RA (N = 2732)	GLP-1RA (N = 1366)	ASD
Age (years)			0.0235
20-39	703(25.73%)	428 (31.33%)	
40-49	852 (31.20%)	374 (27.38%)	
50-59	774 (28.33%)	366 (26.79%)	
60-69	345 (12.62%)	153 (11.20%)	
70-79	38 (1.39%)	36 (2.64%)	
≥80	20 (0.73%)	9 (0.66%)	
Sex			0.0000
Male	1540 (56.40%)	770 (56.40%)	
Female	1192 (43.60%)	596 (43.60%)	
Urbanization			0.0019
1	674 (24.67%)	412 (30.14%)	
2	906 (33.16%)	453 (33.18%)	
3	593 (21.71%)	236 (17.29%)	
≥4	559 (20.46%)	265 (19.39%)	
aDCSI score^#^			0.0556
0	1996 (73.04%)	807 (59.09%)	
1	426 (15.60%)	285 (20.88%)	
2	229 (8.37%)	171 (12.51%)	
3	53 (1.94%)	69 (5.08%)	
4	18 (0.67%)	19 (1.40%)	
≥5	10 (0.37%)	15 (1.05%)	
Comedication			
Biguanides	2244 (82.15%)	1262 (92.41%)	0.0137
Sulfonylureas	844 (30.89%)	581 (42.55%)	0.0624
Alpha glucosidase inhibitors	96 (3.53%)	110 (8.03%)	0.0596
Thiazolidinediones	136 (4.98%)	144 (10.52%)	0.0723
Dipeptidyl peptidase-4	655 (23.97%)	695 (50.86%)	0.0867
Insulin	142 (5.21%)	498 (36.44%)	0.2148*
Statin	1072 (39.24%)	769 (56.27%)	0.0629
Corticosteroids	466 (17.06%)	255 (18.67%)	0.0533

GLP-1RA: glucagon-like peptide 1 receptor agonist, N: number, ASD: absolute standard difference, aDCSI: adapted diabetes complications severity index * Significant difference between the two groups

**Table 2:** Incidence of glaucoma between the GLP-1RA and non-GLP-1RA groups

Event	Non-GLP-1RA group	GLP-1RA group	P
Person-months	116,292	59,078	
Event	91	40	
cHR (95% CI)	Reference	0.768 (0.598-1.059)	
aHR (95% CI)	Reference	0.712 (0.533-0.936)*	0.0025*

GLP-1RA: glucagon-like peptide 1 receptor agonist, aHR: adjusted hazard ratio, CI: confidence interval, DME: diabetic macular edema, PDR: proliferative diabetic retinopathy

**Table 3:** Results of the subgroup analyses of glaucoma development, stratified by age and sex

Subgroup	aHR	95% CI	P for interaction
Age (years)			0.0438*
<60	0.624	0.527-0.921	
≥60	0.759	0.585-0.956	
Sex			0.5621
Male	0.735	0.520-0.942	
Female	0.686	0.543-0.901	

aHR: adjusted hazard ratio, CI: confidence interval, DME: diabetic macular edema, PDR: proliferative diabetic retinopathy * Significant difference between the two groups
